# Supplementary figures and images for: Genome-wide identification and characterization of the fibrillin gene family in Triticum aestivum
Source: PeerJ. 2020 May 26;8:e9225. doi: 10.7717/peerj.9225 (PMC7258936; doi:10.7717/peerj.9225)

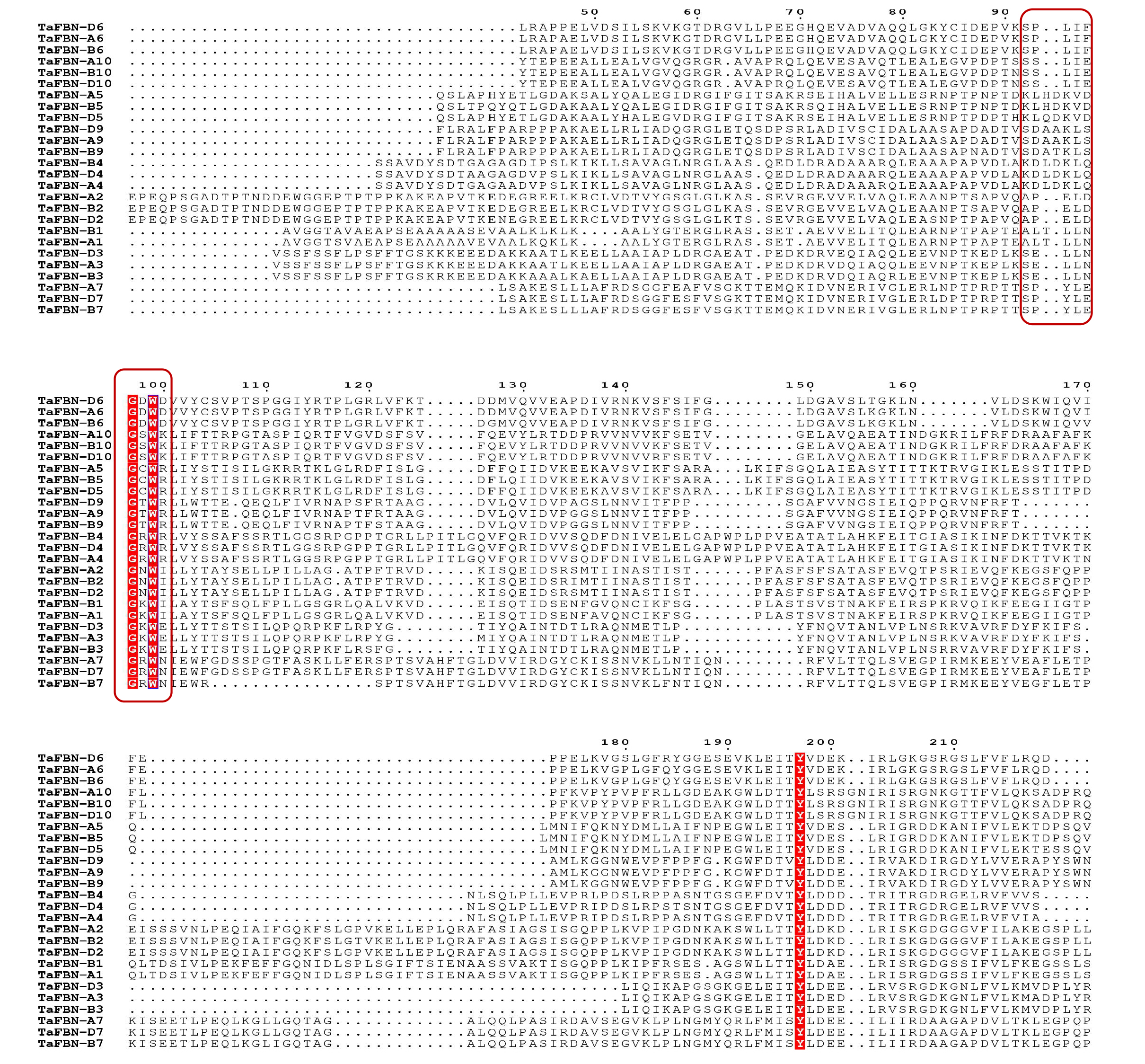

Supplement: Figure S1 — The height of each amino acid code in the sequence logo of each motif represents the degree of conservation. [file peerj-08-9225-s002.jpg]

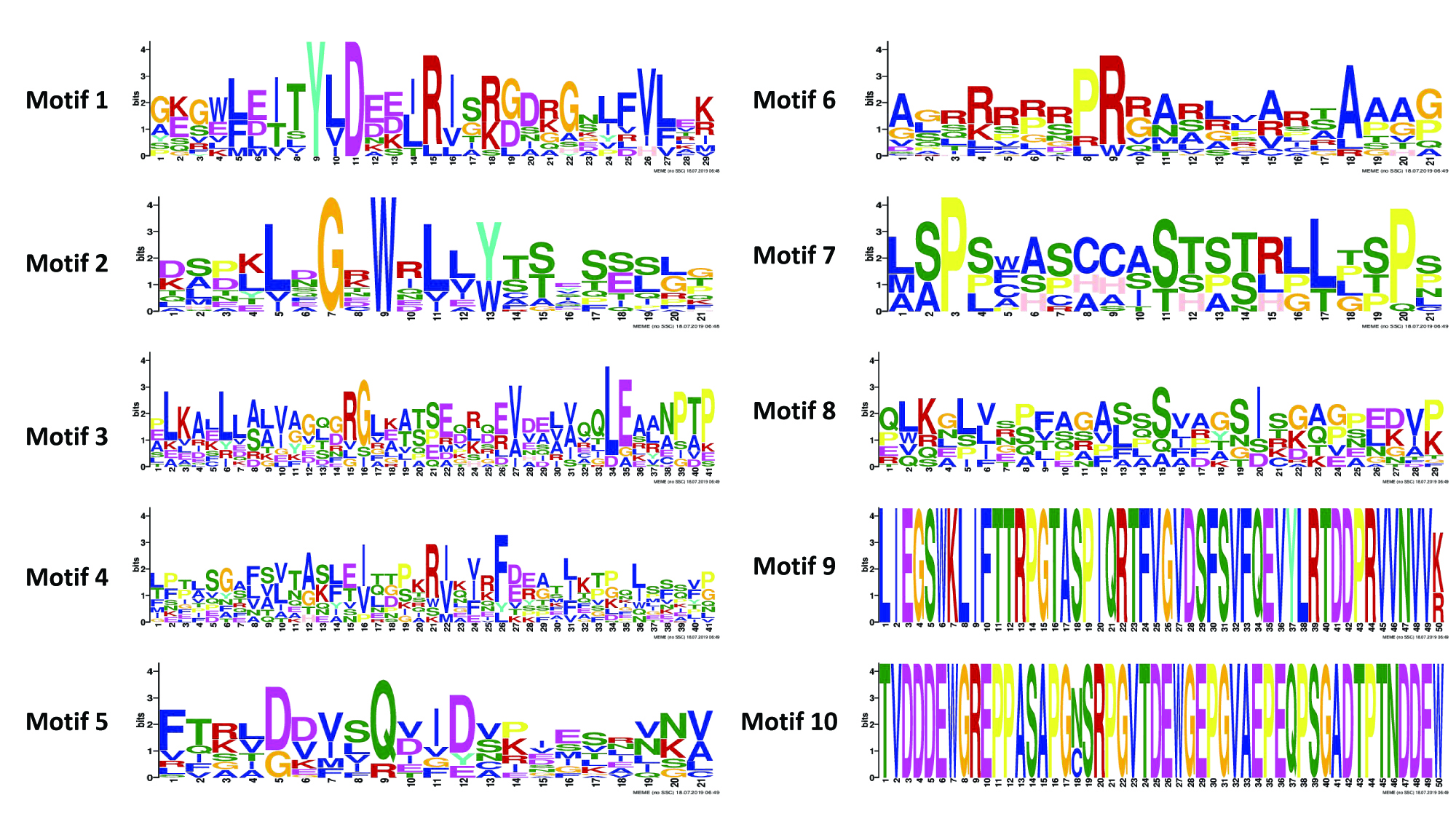

Supplement: Figure S2 — (TaFBN s). Multiple sequence alignment was carried out using clustalw (https://www.genome.jp/tools-bin/clustalw). The red outlined box indicates a conserved lipocalin motif. [file peerj-08-9225-s003.jpg]
